# Supplementary material for: A large-scale evaluation of NLP-derived chemical-gene/protein relationships from the scientific literature: Implications for knowledge graph construction
Source: PLoS One. 2023 Sep 8;18(9):e0291142. doi: 10.1371/journal.pone.0291142 (PMC10490933; doi:10.1371/journal.pone.0291142)
Supplement: S1 File — (DOCX) [file pone.0291142.s001.docx]

# **Supporting information:** A large-scale evaluation of NLP-derived chemical-gene/protein relationships from the scientific literature: implications for knowledge graph construction

Jonathan C G Jeynes^1^, Matt Corney^1^, Tim James^1^

^1^Evotec (UK) Ltd., *in silico* Research and Development, 114 Innovation Drive, Milton Park, Abingdon, Oxfordshire OX14 4RZ, United Kingdom

**Author Information**

Corresponding Authors

*E-mail: [charlie.jeynes@evotec.com](mailto:charlie.jeynes@evotec.com)

**Short title:** NLP derived chemical-protein relationships


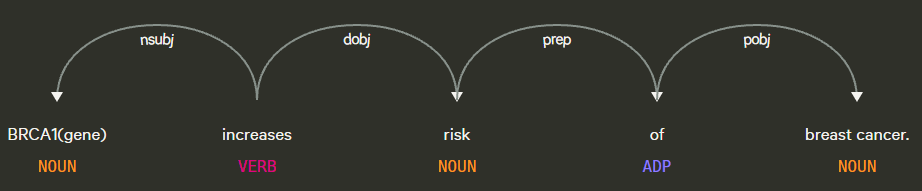


S1 Fig. An example of dependency parsing. A language model is used to tag each word in the sentence with a Part-of-Speech (e.g. noun, verb, adjective, etc), and a dependency path is derived (e.g. nsub, dobj, etc). For the GNBR dependency paths, an additional ‘pruning’ stage was also included as explained in Percha *et al*. 2018. Abbreviations: “ADP” = adposition, “nsubj” = nominal subject, “dobj” = direct object, “prep”= preposition, “pobj” = object of preposition (a detailed explanation is found Manning *et al*. 2013). This example was made using the Spacy dependency visualizer (https://explosion.ai/demos/displacy).


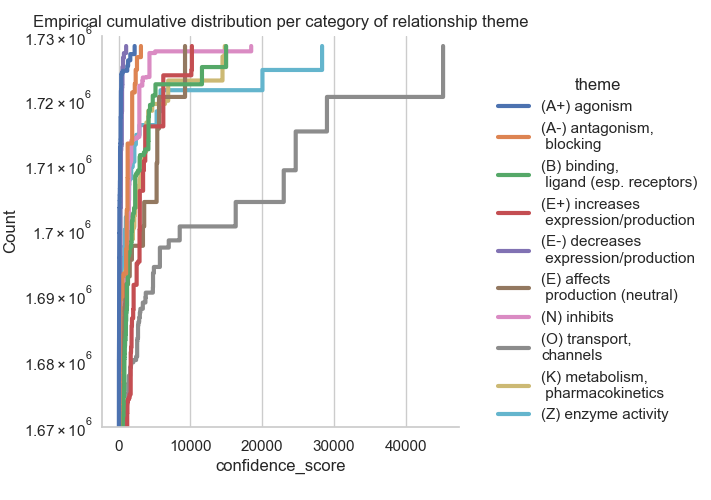


S2 Fig. Empirical cumulative distribution per GNBR theme. This shows that most scores in GNBR are between 1-100, while there are a small minority of scores which range from 100-40,000, depending on the theme.


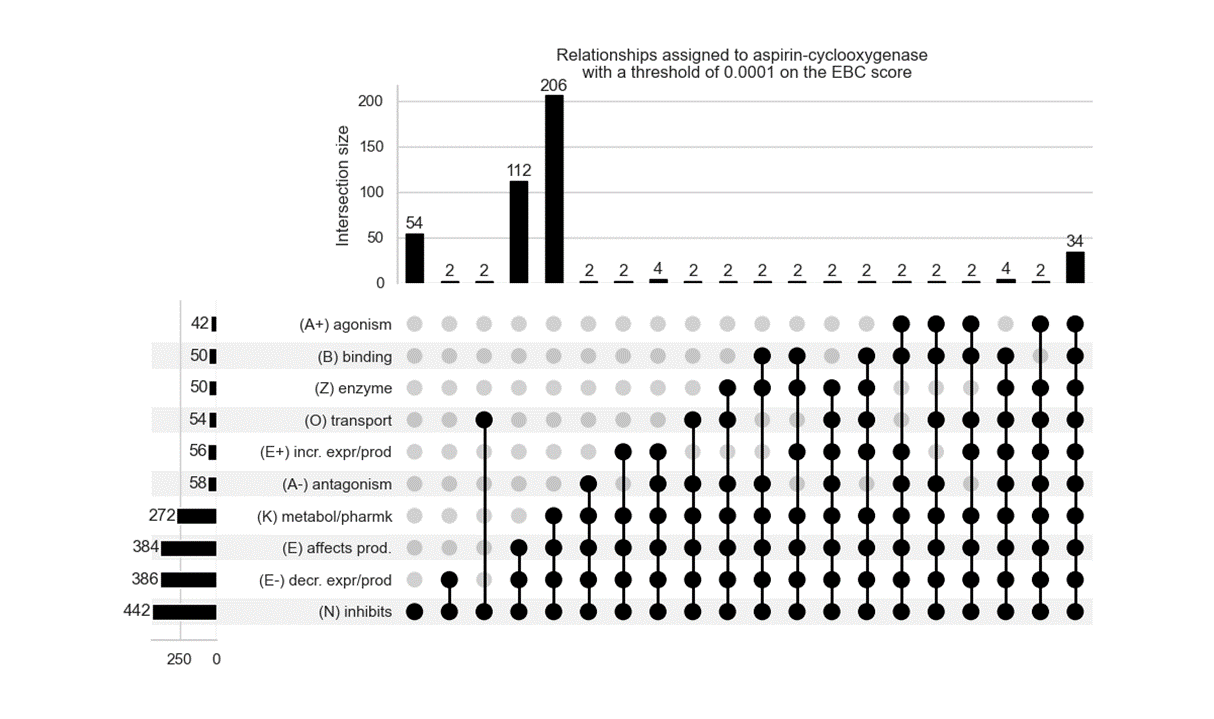


S3 Fig. An upset plot showing the relationships assigned to 442 sentences with a threshold of >0 where ‘aspirin’ and ‘cyclooxygenase’ are the chemical and protein entities. An upset plot can be read as follows: the left-hand bars show the total number of sentences which had any given category as true. So here, all 442 sentences had ‘(N) inhibits’ as true. The top-most bars show sentences which have the categories indicated with a black dot as true. Here, 54 sentences are predicted to exclusively have the relationship ‘(N) inhibits’, 206 sentences fall in four categories, while 34 sentences are weighted across all 10 relationship classes.


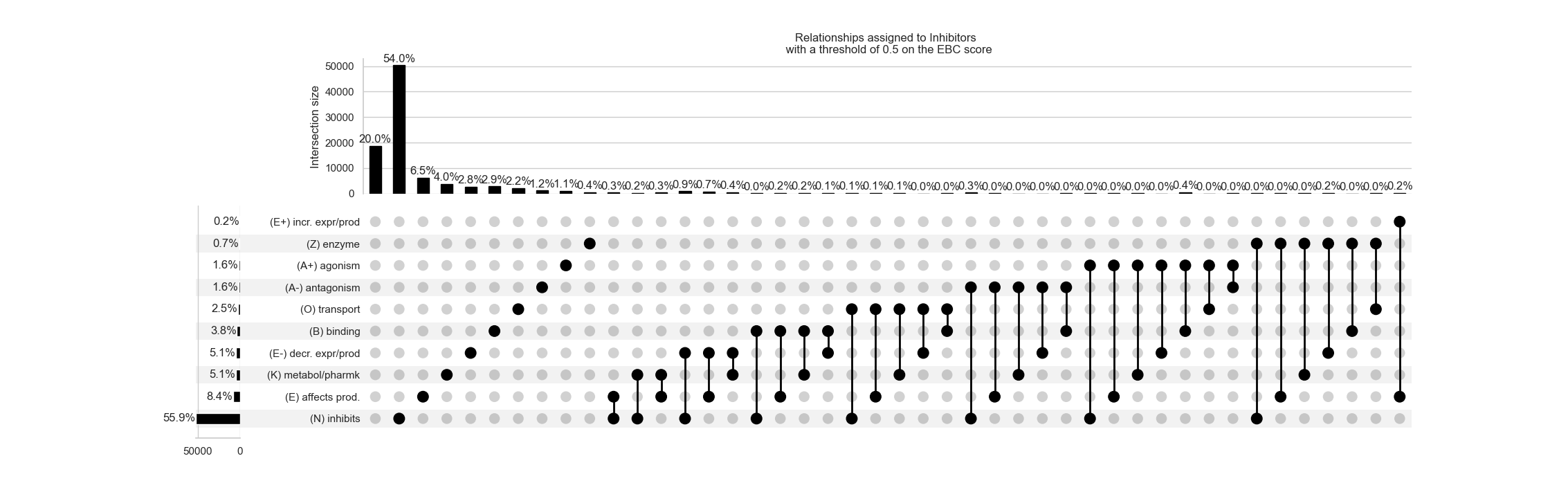


S4 Fig. A visualization of how GNBR categorises chemical-protein relationships compared to Nexus’ Inhibitors class at a threshold of 0.5. We plot the percentage of sentences that fall into the GNBR relationship themes or do not have a high enough score for any category (i.e. the 20.0% bar).
